# Characterizing social-ecological context and success factors of antimicrobial resistance interventions across the One Health spectrum: analysis of 42 interventions targeting *E. coli*

**DOI:** 10.1186/s12879-021-06483-z

**Published:** 2021-08-26

**Authors:** Anaïs Léger, Irene Lambraki, Tiscar Graells, Melanie Cousins, Patrik J. G. Henriksson, Stephan Harbarth, Carolee A. Carson, Shannon E. Majowicz, Max Troell, E. Jane Parmley, Peter S. Jørgensen, Didier Wernli

**Affiliations:** 1 Global Studies Institute, University of Geneva, Sciences II, Quai Ernest-Ansermet 30, Case postale, 1211 Geneva, Switzerland; 2 School of Public Health and Health Systems, University of Waterloo, 200 University Avenue West, Waterloo, ON N2L 3G1 Canada; 3 Global Economic Dynamics and the Biosphere, The Royal Swedish Academy of Sciences, Box 50005, 104 05 Stockholm, Sweden; 4 Stockholm Resilience Centre, Stockholm University, Kräftriket 2B, 10691 Stockholm, Sweden; 5 Beijer Institute of Ecological Economics, The Royal Swedish Academy of Sciences, P.O. Box 50005, 104 05 Stockholm, Sweden; 6 WorldFish, Jalan Batu Maung, 11960 Bayan Lepas, Penang Malaysia; 7 Infection Control Program and WHO Collaborating Centre on Patient Safety, University of Geneva Hospitals and Faculty of Medicine, Geneva, Switzerland; 8 Centre for Food-Borne, Environmental and Zoonotic Infectious Diseases, Public Health Agency of Canada, Guelph, Canada; 9 Department of Population Medicine, Ontario Veterinary College, University of Guelph, 50 Stone Road East, Guelph, ON N1G 2W1 Canada

**Keywords:** AMR, Social-ecological system, Implementation science, Enterobacteriaceae, *E. coli*

## Abstract

**Background:**

Antimicrobial resistance (AMR) is among the most pressing One Health issues. While interventions and policies with various targets and goals have been implemented, evidence about factors underpinning success and failure of interventions in different sectors is lacking. The objective of this study is to identify characteristics of AMR interventions that increase their capacity to impact AMR. This study focuses on AMR interventions targeting *E. coli*.

**Methods:**

We used the AMR-Intervene framework to extract descriptions of the social and ecological systems of interventions to determine factors contributing to their success.

**Results:**

We identified 52 scientific publications referring to 42 unique *E. coli* AMR interventions. We mainly identified interventions implemented in high-income countries (36/42), at the national level (16/42), targeting primarily one sector of society (37/42) that was mainly the human sector (25/42). Interventions were primarily funded by governments (38/42). Most intervention targeted a low leverage point in the AMR system, (36/42), and aimed to change the epidemiology of AMR (14/42). Among all included publications, 55% (29/52) described at least one success factor or obstacle (29/52) and 19% (10/52) identified at least one success factor and one obstacle. Most reported success factors related to communication between the actors and stakeholders and the role of media, and stressed the importance of collaboration between disciplines and external partners. Described obstacles covered data quality, access to data and statistical analyses, and the validity of the results.

**Conclusions:**

Overall, we identified a lack of diversity regarding interventions. In addition, most published *E. coli* interventions were poorly described with limited evidence of the factors that contributed to the intervention success or failure. Design and reporting guidelines would help to improve reporting quality and provide a valuable tool for improving the science of AMR interventions.

**Supplementary Information:**

The online version contains supplementary material available at 10.1186/s12879-021-06483-z.

## Background

Antimicrobial resistance (AMR) is a pressing global issue in human and animal health and is frequently included as a priority in political agendas at national and international levels. AMR is a natural phenomenon that has been accelerated by the broad use of antimicrobials (AM) in modern medicine and the food producing system. Development of multi- and pan-resistant bacteria decreases our capacity to treat common infections, and increases the burden of such infections in terms of lives lost and financial costs. Over the past ten years, efforts to tackle AMR have increased worldwide. For example, the healthcare sector adopted tailored stewardship programmes and guidelines for antimicrobial use (AMU) [1] and strengthened surveillance [2]. Actions in animal production include voluntary industry bans regarding the use of specific AM, such as the ceftiofur withdrawal in the broiler industry in Japan [3] and Canada [4, 5]. Since the adoption of the World Health Organization (WHO) Global Action Plan in 2015, many countries have developed national actions plans, built coordinated national actions, identified targets for interventions and described expected benefits for human health [6]. Efforts are also being made to develop a One Health approach by integrating AMR and AMU data from several sectors [7, 8]. Some integrated programmes have identified several different actions that, when implemented together, increase the potential for successful reduction of AMR [9]. All these efforts are reflected in the growing number of interventions being reported in the literature.

In a previous publication, we defined an intervention as a coordinated action driven by a social group among a targeted population in a bio-ecological context that interferes with the outcome or course of a difficult situation or process in order to improve it or prevent it from getting worse [10]. The term covers a variety of actions, related to the trigger and goal of the intervention, as well as resource availability, context of application, and more. Interventions can also be called initiatives, actions, programmes, and incentive strategies.

AMR issues are the result of complex interactions in tightly coupled social-ecological system (e.g., animal husbandry system, AM prescription habits, food consumption, and sanitary levels) and should therefore be designed according to their context to maximise impact [11]. While interventions often target a single factor, the goals is to influence the broader AMR social-ecological system. How they affect the system depends on their aim, governance, targeted pathogen, and other factors. Hence, implementation science, the study of methods and strategies that enhance the translation of evidence-based practice and research into usable material for practitioners and policymakers, is of critical importance [12, 13]. Implementation science provides insights about previous experiences and, as this knowledge grows, aims to identify patterns of intervention success (and failure). This, in turn, can be used to improve our understanding of the factors that contribute to the success or failure of interventions. Some studies have started to identify criteria for implementation of One Health interventions for AMR, e.g., action oriented surveillance systems [14–16]. Nevertheless, more evaluations are needed to support success of all actions to address AMR.

The need for systems thinking to support description, study, and evaluation of complex issues such as AMR has been acknowledged in previous publications [11, 17]. A perspective that stresses the importance of a dynamic, systems approach [18] and recognizes the nonlinear, multi-component and context-dependent nature of interventions can help improve how we address AMR. In the recently published AMR-Intervene framework [10], we outlined how AMR interventions can be used to build societal resilience to AMR by implementing actions that influence AMR from its drivers to its impact [10, 19]. The AMR-Intervene framework is organised into six components: (i) core information about the publication; (ii) social system; (iii) bio-ecological system; (iv) triggers and goals; (v) implementation and governance; and (vi) assessment of the intervention. The framework aims to capture the specific context (e.g., practicality, implementation, acceptability, actors, other interventions implemented, AMR situation) and the broader context (e.g., national AMR governance, level of development of the country) in which the intervention was implemented.

The objective of this narrative review is to identify characteristics of AMR interventions that affect their implementation in social-ecological AMR systems, focusing on interventions targeting resistant *Escherichia coli* (*E. coli*). Resistance in gram-negative bacteria, such as *E. coli*, has been identified as one of the most pressing AMR issues [20, 21]. *E. coli* is a potential pathogen that is frequently identified in humans and animals and can survive for a long time in the environment. Also, *E. coli* is considered a good indicator of resistance levels in the community. This study is part of the AMResilience (https://amr-resilience.gtglab.net/) project, which aims to provide and validate a comprehensive multi-method assessment of resilience and transformability to limit AMR and AMU in national and regional One Health systems [22]. Here we aim to examine the factors that affect or contribute to intervention success. Enhancing the description of AMR interventions and their social-ecological systems will help increase general knowledge to better design and implement effective AMR interventions.

## Methods

### Identification of *E. coli* AMR interventions

Within the scope of the AMResilience project, we set up a general database of published AMR interventions based on a scoping review of the literature. We used a Boolean query with search strings validated among the co-authors (Additional file 1, Additional file 1: Table S1), and searched the PubMed online database in June 2018 (Additional file 1: Table S1). As the number of results was large (more than 26,000 records), we narrowed the search to include only literature reviews by adding “publication type” search terms. We screened the publications in three steps: title review, abstract review, and article review (Additional file 1: Table S2). The first author conducted the review with support from the last author.

The title review followed the inclusion and exclusion criteria described in Additional file 1: Table S3. Publications were excluded when: (i) the title did not state at least one of the following terms: antimicrobial, antibiotic, drug, or resistance; and (ii) the title clearly indicated that the article did not focus on an AMR intervention (e.g., policy comparison papers, recommendation papers, and guidelines). We conducted a title review on all references within the review papers and included additional publications in the pool of documents for the abstract review. Any duplicate articles were excluded.

All retained articles underwent an abstract review using the inclusion and exclusion criteria described in Additional file 1: Table S3. Articles were excluded when (i) abstracts were not written in English or French (i.e., languages fluently read from the first and last authors); (ii) abstracts described theoretical studies with no empirical data presented; and (iii) any of the exclusion criteria of the title review that were not apparent from reading the title only. At this stage, we shared the list of AMR interventions among the co-authors and asked for relevant interventions or reviews of AMR interventions of their knowledge that were not included.

The article review involved reading and screening the full articles, based on inclusion and exclusion criteria described in Additional file 1: Table S4. Articles referring to AMR interventions were selected with no limits based on the intervention quality, or study type or quality. Instead, the AMR-Intervene database recorded criteria based on the article’s description and assessment of the intervention [10].

To avoid missing publications about *E. coli,* we conducted a new online search specifically targeting *E. coli* AMR interventions in June 2019 (Additional file 1: Tables S1, S2), following the same process as in June 2018. While not exhaustive, this database provides a good basis for evaluating the literature on AMR interventions that specifically target *E. coli*. A populated checklist from the Prisma-ScR is available for this study (Additional file 4).

### Analyses of *E. coli* AMR interventions

We used the AMR-Intervene framework to code all identified documents referring to *E. coli* AMR interventions. This work was completed between September 2019 and March 2020. The first author coded each publication and the last author coded two randomly selected publications for comparison to ensure consistency in coding. Problematic coding was discussed with the last author until consensus was reached.

AMR-Intervene is based on an online form built upon a set of questions to facilitate the coding process. Most of the values of the categorical variables used to characterize an intervention were not mutually exclusive. The form provides a coder with a drop-down menu and multiplechoice questions for many of the variables, and allows the addition of new values for each question that are not captured by the current framework. Thus, it is possible to incrementally improve the framework and capture unexpected data. For articles referring to the same intervention, data were merged to conduct analyses by intervention rather than by publication, after checking for consistency. The last part of the AMR-Intervene framework, i.e. assessment, is organized in open questions to capture key results, success/failure factors, and unexpected consequences of any intervention. Thus, the framework enables coding of both qualitative and quantitative data.

Descriptive analyses of data from *E. coli* AMR intervention articles were conducted in R [23]. We extracted simple information such as mode, median, minimum, and maximum; and computed graphical representation of the data for all relevant variables in the database. Investigating the distribution of data allowed us to capture the diversity of the interventions as well as the most frequent characteristics among all studied interventions. Thus, we were able to capture a typical intervention based on the most representative features of implementation and organisation.

Coding of text from the open-ended questions in the AMR-Intervene was conducted using qualitative analysis software (ATLAS.ti 8 Windows, Scientific Software Development GmbH), referencing the text used for each coding. The first author then conducted a thematic analysis of the text extracts following the classification of the framework for advancing science Consolidated Framework for Implementation Research (CFIR) [24], but coding was not limited by this framework if other themes emerged. This framework gathers criteria of implementation theories using homogenous terminology and aims to facilitate the identification of factors and constructs that enhance success across multiple contexts.

## Results

### Descriptive analyses of coded articles

Our review of scientific papers identified 52 publications describing and assessing *E. coli* AMR interventions (Additional file 1). From the 52 publications selected for this review, 42 different *E. coli* AMR interventions were identified, with four interventions mentioned in two to five papers (Additional file 3, Additional file 2: Table S1 and Additional file 3: Figure S1). The coded articles were mainly published after 2015 (Additional file 2, Additional file 2: Figure S2). In Table 1, we detailed the typical *E. coli* AMR intervention, i.e., summary of the most common characteristics from the 42 interventions coded using the AMR-Intervene framework.

**Table 1:** Summary of the main characteristics identified from studying the 42 interventions coded to highlight the main features of the interventions for each building block in the AMR-Intervene database with the smallest/most narrow, largest/most broad, and most common value for selected variables of AMRIntervene

Variable	Minimum	Typical intervention	Maximum
Start year of the intervention	1982	2004	2012
Duration of the intervention	1 year (7/42)	Ongoing intervention when assessed (27/42)	7 years (1/42)
Country of implementation	One country (40/42)	One HIC (36/42)	Several countries involved (2/42)
Level of implementation	One level of implementation (37/42)	National (16/42)	Several levels involved (5/42)
Sector	One sector of implementation (human or animal, 37/42)	Human sector (25/42)	Several sector involved (not frequent, 3/42)
Social system	No specific social group targeted (26/42)	No specific social group targeted (26/42)	Several social groups targeted (29/42)
Number of bacteria targeted	No specific bacteria targeted (13/42)	Enterobacteriaceae (29/42)	Three bacteria targeted (3/42)
Number of AM targeted	No specific AM classes targeted (9/42)	Betalactams (26/42)	7 AM classes targeted (3/42)
Responsible	One institution responsible (28/42)	Governmental institution (21/42)	Partnership (2/42)
Funding source	Privately funded (3/42)	Government funded (38/42)	Partnership (1/42)
Type of policy instrument used by the intervention	One type of policy instrument (29/42)	Regulation (19/42)	Two types (7/42)
Trigger	On trigger (31/38)	State (15/42)	3 different types of trigger (1/42)
Goal	One goal (28/42)	State (16/42)	3 different types of goals (2/42)
Challenge of collective action	One challenge (34/42)	Surveillance (18/42)	3 different types of challenges (1/42)
Leverage point of the intervention		Low leverage point (36/42)	
Number of key results assessed	One key result (6/52)	3 key results assessed (16/52)	Five or more key results (4/52)

The table gathers the main characteristics identified from studying the 42 interventions coded to highlight the main features of the interventions for each building block in the AMR-Intervene database with the smallest/most narrow, largest/most broad, and most common value for selected variables of AMRIntervene

**Fig. 1:**
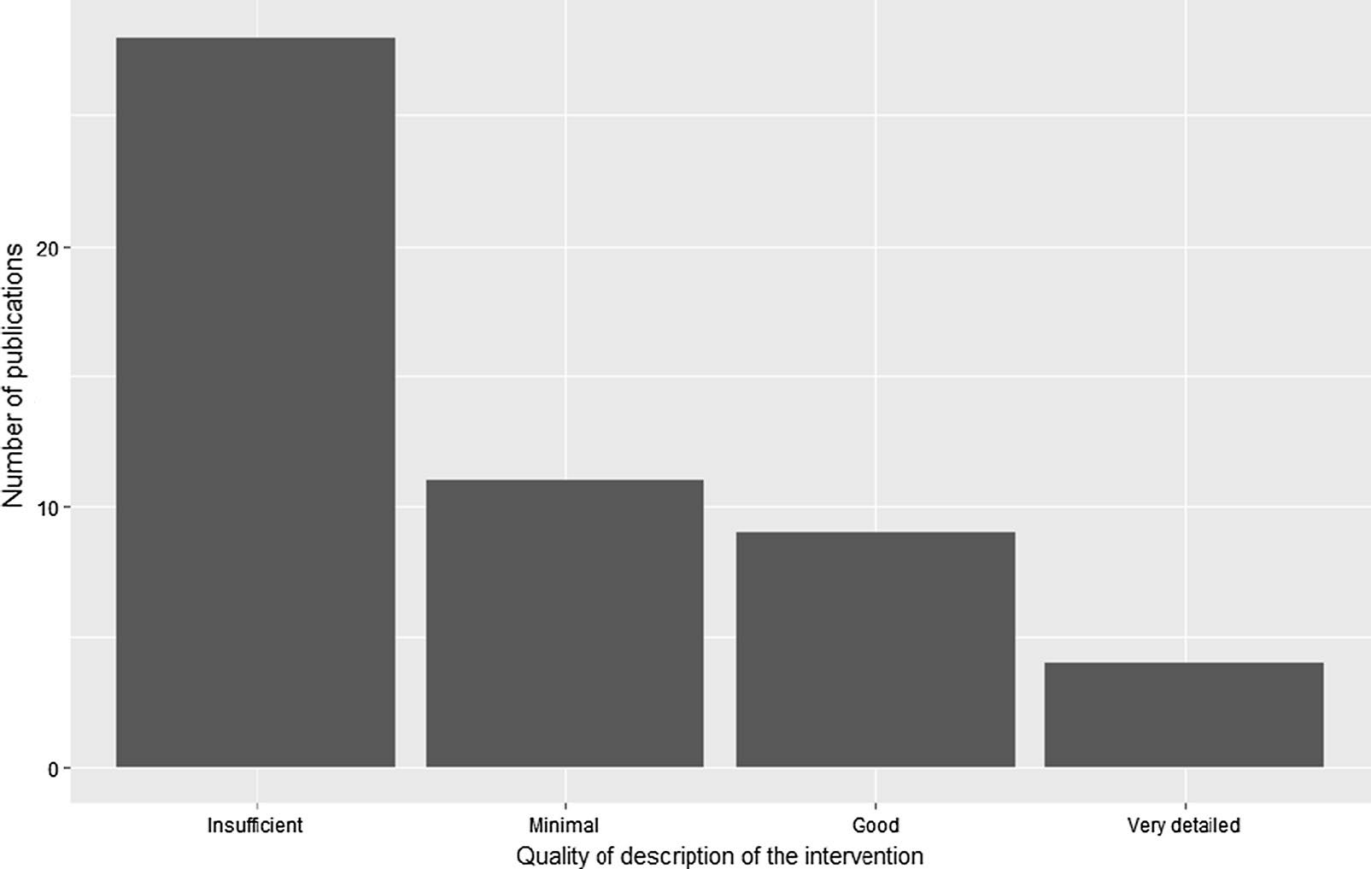
Results of the qualitative assessment of description of the intervention in the scientific publications (n = 52 articles coded)

Based on the qualitative scoring system used in the AMR-Intervene framework, most interventions were poorly described; 28 documents provided insufficient detail, 11 only provided minimal details, and 3 did not provide any assessment of the intervention they described (Fig. 1). The study designs for intervention evaluation were mainly cross-sectional (26/52), narrative (7/52), cohort (4/52), time series analyses (3/52), and reviews (3/52). Objectives of the evaluations were not clearly defined in 3 of the 52 publications. Forty-two (42/52) studies used quantitative data for assessment, while the others relied on qualitative assessment. Assessment of interventions was mainly conducted when the intervention was still in progress (31/52).

**Fig. 2:**
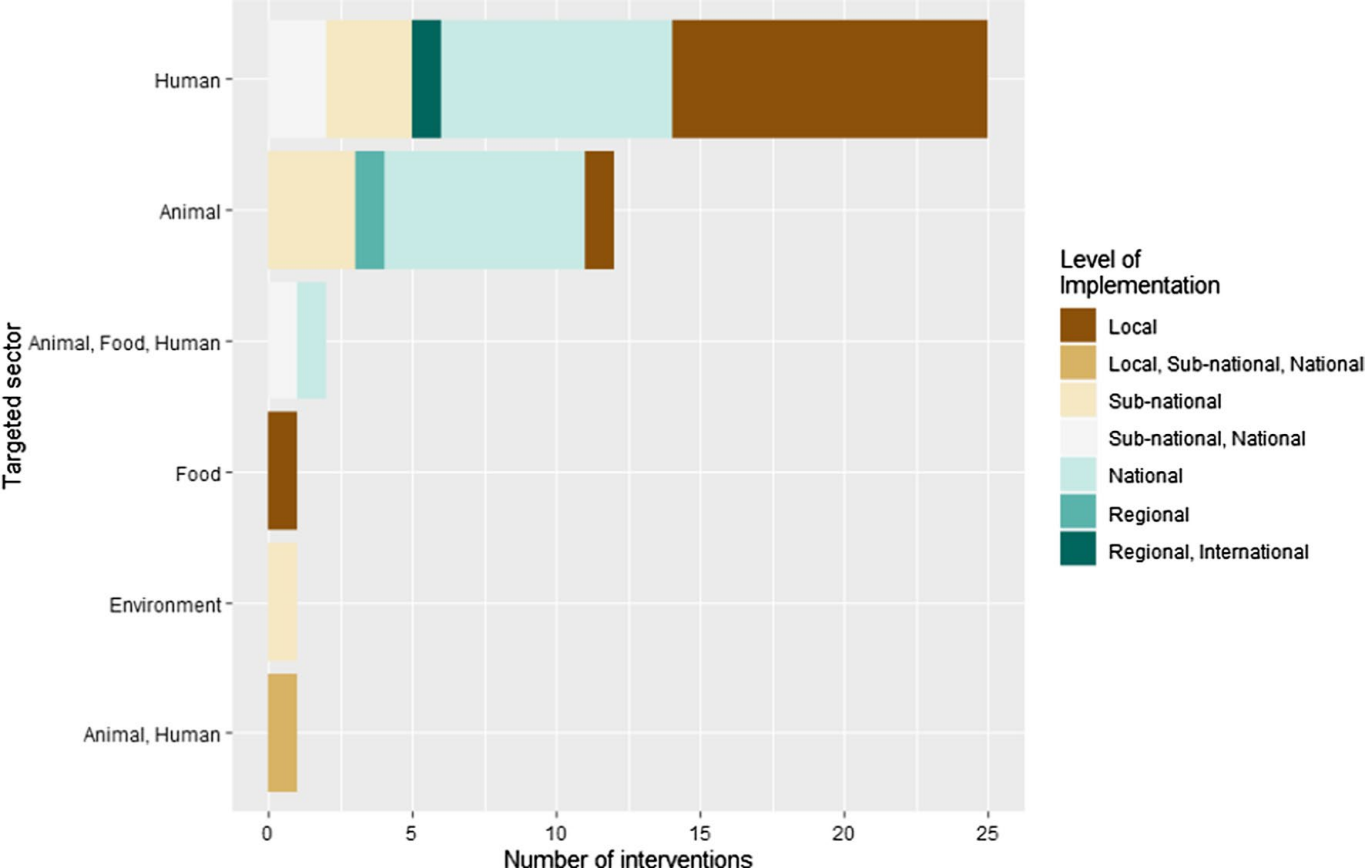
Targeted sector of the *E. coli* AMR interventions depending on their level of implementation (n = 42 *E. coli* AMR interventions from 52 articles coded)

Only 2 of the 42 interventions were implemented in more than one country. All others were implemented in a single country, most frequently the USA (5/42), Canada (4/42), the Netherlands (4/42), China (3/42) United Kingdom (3/42), and Denmark (3/42). Among the coded interventions, 36 targeted high-income countries, 4 middle high-income countries and 1 a low-income country. The interventions were implemented from 1982 to 2012 (Additional file 3: Figure S2). We mainly identified interventions targeting only one sector, either animals (12/42) or humans (25/42) (Fig. 2). Interventions targeting humans were predominantly focused on healthcare delivery (24/28) and the general population (2/28). Interventions targeting the animal sector primarily focused on livestock (13/15). Three (3/42) interventions targeted food including meat (2/3) and vegetables (1/3). Only one (1/42) intervention targeted the environment, focusing on wastewater. Most interventions were implemented at the national (16/42), or the local/subnational level (20/42). For the most part, the *E. coli* AMR interventions targeting the animal or human sector had no specific social group targeted, i.e., they focused on the general population. The interventions were implemented in different settings according to the sector targeted. Interventions in animal health were implemented at farms (3/15), slaughterhouse (3/15) or slaughterhouse and farm (3/15), or no specific setting (6/15). Interventions targeting human health were mainly implemented in hospitals (21/28). Interventions targeting food mainly targeted supermarkets (2/3).

Among the coded interventions, 29 targeted Enterobacteriaceae in their activities, but not exclusively: 7 targeted *E. coli* only, 12 targeted other gram positive bacteria, and 10 targeted other gram negative bacteria. Thirteen interventions targeted no specific bacteria; resistance in *E. coli* was used as a measure of outcome. Interventions usually targeted one class of AM (median = 1), but the maximum was seven AM classes (Additional file 3: Figure S3). The main targeted AM classes were beta lactams (26/42), quinolones (20/42), and aminoglycosides (15/42), followed by tetracyclines (12/42) and sulphonamides (10/42). Most interventions did not focus on a specific syndrome or disease (33/42), while seven focused on bloodstream infections, six on respiratory diseases (lower and upper tract), and four on urinary tract infections.

**Fig. 3:**
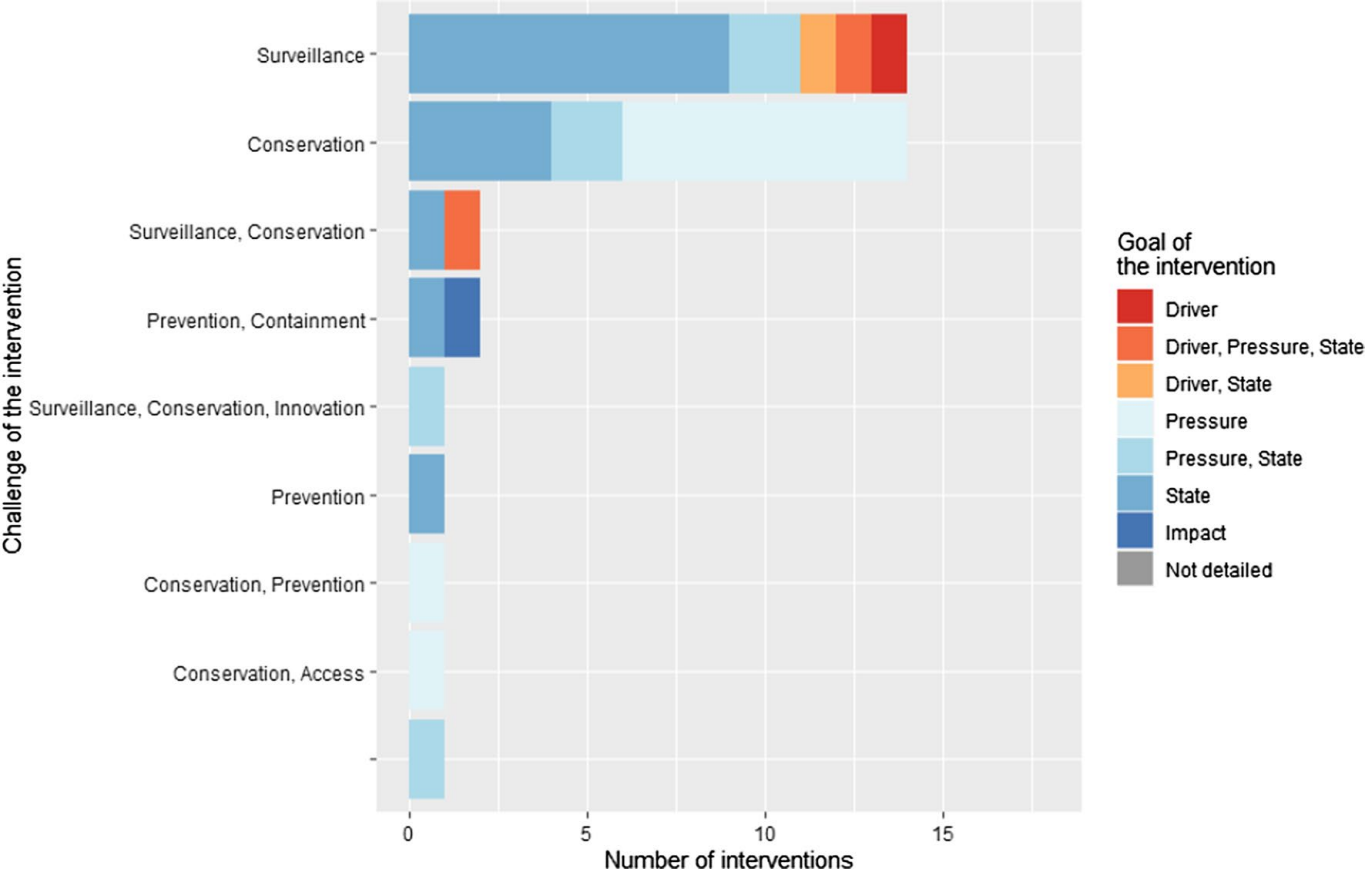
Challenge of the implementation of the *E. coli* AMR intervention in relation to the goal of the intervention (n = 42 *E. coli* AMR interventions from 52 articles coded)

**Fig. 4:**
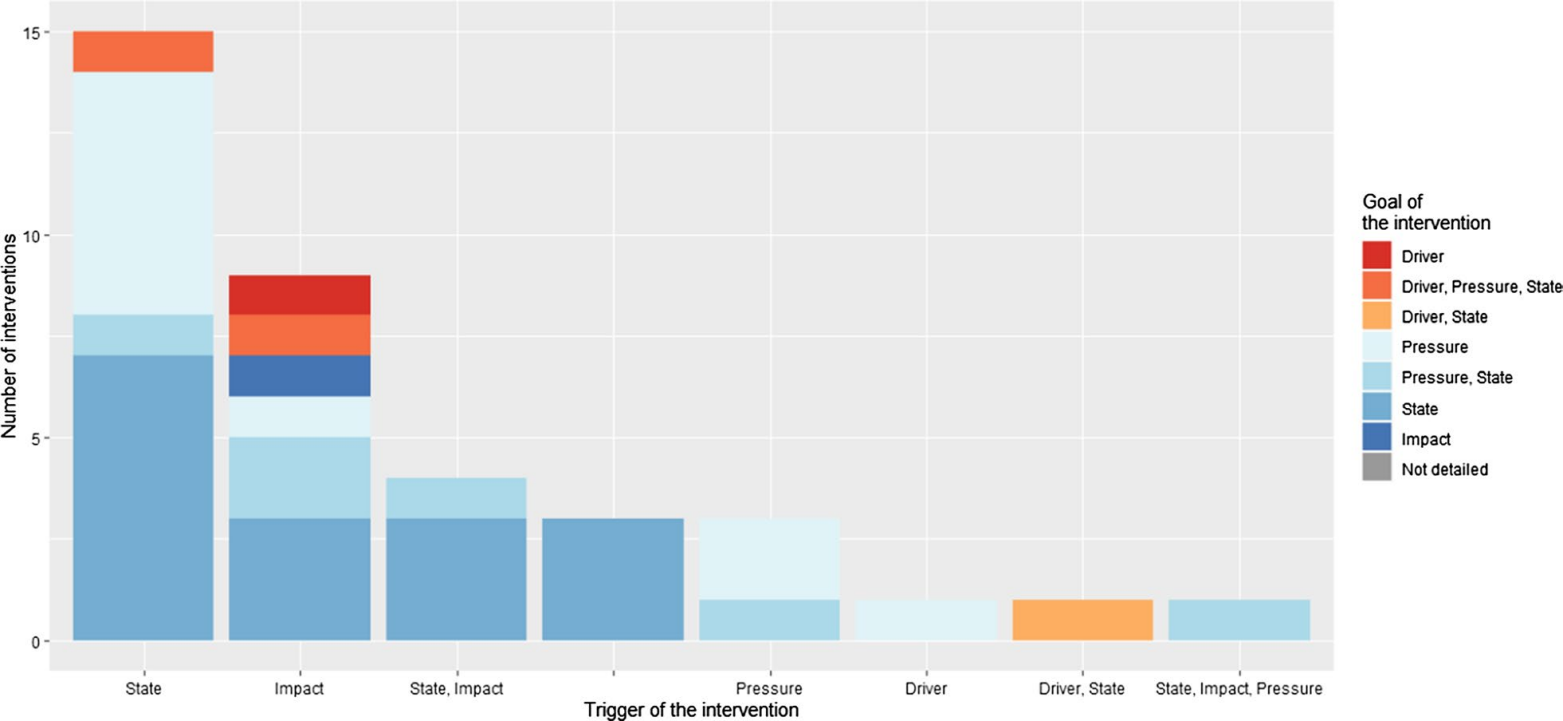
Trigger of the *E. coli* AMR interventions in relation to the goal of the intervention (n = 42 *E. coli* AMR interventions from 53 articles coded)

Most coded interventions described specific triggers for the intervention (38/42). Most interventions had one specific trigger (31/38), usually the epidemiologic state of AMR (15/38) or the health impact of AMR such as the morbidity/mortality (12/38) (Fig. 4). In Fig. 3, the main policy priorities addressed by the interventions were surveillance (21/42) and conservation of antimicrobials (20/42). The goals of the interventions were mainly to reduce the state of AMR (i.e. the prevalence of drug-resistant pathogens) (25/42) and AMU (18/42). Among all the interventions, 34 promoted positive action towards AMR control (i.e. to do something right or better), while eight interventions were about restricting or prohibiting something. Finally, among all coded interventions, many targeted a low leverage point in the AMR chain, focused on changing some parameters of the system without addressing the more distal drivers of the emergence and transmission of AMR (36/42).

The main entities responsible for implementation were governments (23/42), some in collaboration with private partners (2/23), followed by research-based organisations (6/42) and private organisations (3/42); also shown in Additional file 3: Figure S4. Governments were the principal source of funding supporting AMR interventions (38/42) (Additional file 3: Figure S5). Only two interventions clearly mentioned the level/amount of funding for the intervention. Among the interventions and their governance instruments, most used only one type of policy instrument (29/42), mainly using an information strategy (17/42, i.e., encouraging people to act or do something by providing information) and regulation (12/42, i.e., requiring people to do something with potential penalty in case of no compliance). Only seven interventions used two different types of regulations when implementing the activities. Ten interventions (10/42) used self-regulation to address AMR. A clear description of all actors involved in the design and implementation of intervention was only accessible in six publications (6/52), varying from 1 to 56 different actors.

Among reported key results, we identified 101 positive results towards AMR (out of 137 key results reported in total). Key results can be identified as 104 outputs of the interventions and 33 outcomes. Among all key results, we identified 3 related to the drivers of AMR, 37 related to the pressure of AMU towards AMR, 59 related to the state of AMR, 5 related to the impact of AMR, and finally 32 directly related to the intervention and its delivery, implementation or success.

Among all publications, 13% (7/52) mentioned the identification of positive and negative unintended consequences and six (6/7) described them. The identified unintended consequences can be organised in three categories. First, the intervention enabled hiring of more medical professionals as well as specialists in AM treatments in a hospital. Second, the intervention enhanced a shift in the use of AM from one class to another. Finally, issues related to the duration of an intervention’s effect suggests that the observed reduction of AMU might not be permanent.

### Success and failure factors

Among all coded documents, 55% of the publications described at least one success factor or obstacle to intervention implementation (29/52). Among them, 10 publications (19%, 10/52) explained at least one success factor and one obstacle. All factors are detailed in Table 2 and additional details of the success factors and obstacles are provided in Additional file 3: Table S6. Sixteen of the coded publications described contextual events or factors that were outside the control of implementers that positively or negatively impacted the outcome(s) of the intervention, for example, a concomitant event or a cultural habit.

**Table 2:** Details of success and failure factors and contextual factors identified in the 52 studied publications

Main domain of CFIR classification	Subdomain of CFIR classification	Description of the factor (success, failure, or contextual factor)
Interventions characteristics	Importance of evidence strength and quality	Success factor: A proper strategy for data access, management, and analysis was identified as a success factor. Data should also be checked for plausibility and completeness
		Failure factors: Several publications highlighted data limitations as potential failure factors of their intervention, including small number of data/samples, quality of the data (e.g., sampling, recording, entering the data in the database, consistency of data recording, bias in data collection, quality control, semantic homogeneity), low representativeness of the data, and the need for long-time data Some authors also mentioned the lack of adequate literature and knowledge to help them conducting their research
	Adaptability	Success factor: Interventions were reported more successful if individually tailored regarding their features and methods while remaining flexible to adapt along with the intervention lifecycle. Activities that were adapted or changed as needed during their lifecycle were more likely to be accepted, useful, and to successfully lead to intended outcomes. For instance, some interventions changed AMU guidelines, expanded the scope of a surveillance system, and conducted or recommended that interventions be evaluated throughout their lifecycle
	Complexity of intervention	Success factors: Multimodal interventions were also reported to increase the success of interventions. For example, one activity such as surveillance of AMU in animals can be supported by several others like guideline implementation, awareness campaigns, group discussions, and media dissemination. Interdisciplinary teams and collaboration between disciplines was identified several times as a catalyst for success. It has been suggested as a factor of success but also an empowering factor for achieving goals. Early involvement of all actors (e.g., stakeholders, multidisciplinary actors) was another supportive action for the intervention Decentralising actions to regional and local groups with a diversity and multiplicity of actors was considered to increase the success of the interventions. Also, embedding the intervention in pre-existing structures and using existing resources (e.g., sampling strategy already in place) and collaboration with local scientific and political partners may increase the success of the intervention
	Costs	Success factor: Interventions were identified as costly and require a sustainable financial support to access data and maintain activities
Outer and inner setting	Networks and communication	Success factors: The communication with the general public, sometimes with the collaboration with media, was a real asset for intervention to increase acceptability but also visibility, transparency, and support Communication within the intervention team and actors was important to encourage the implication and compliance to the intervention. Feedback from actors was also necessary for adapting the intervention if its design did not fit its purpose Collaboration and participation
	External policies and incentives	Contextual factors: Interventions at the national level that were implemented concurrently with the studied intervention made an independent assessment of the intervention’s impact difficult, and it might have increased its acceptability/compliance within the target population (e.g., surveillance system and vaccination programme) Access to healthcare was found to have an influence in the access of AMs, their use, and willingness of people to be treated (e.g., lack of health insurance policy)
	Culture	Contextual factor: A commitment for AMR control in the country was found to make an intervention better accepted by the actors and targeted population. However, a track record of actions against AMR was also suggested to lead to smaller impact of the intervention, as the previous interventions already improved the situation regarding AMR in the country. This might reflect the diminishing return of some action with a low leverage point. Nonetheless, having functioning institutions in a state, which ensures compliance with interventions, is an essential cornerstone for limiting AMR
Process	Engaging	Failure factor: The lack of results was also related to the lack of incentives to participate and follow instructions of the intervention
		Success factors: Direct and strong support from the hierarchy was mentioned as a factor to enhance success of the intervention by providing leadership and support. Assigning clear responsibilities for the intervention can also contributed to successful intervention implementation. The funding of a specific coordinator or assistant within the intervention was also described as important
	Threshold for intervention effectiveness	Failure factor: Threshold for intervention effectiveness reached was identified in one publication which mentioned that the intervention implemented in the country could have reached the limits of effectiveness

Details of success and failure factors and contextual factors that had an impact on the interventions as described in the 52 studied publications, following the classification of the CFIR framework (Consolidated Framework for Implementation Research) developed by Damschroder et al. [24]

Success and failure factors included the intervention characteristics (i.e., details about its implementation and organisation), the outer and inner settings of the intervention (i.e., changes in context that can influence the intervention), and the implementation process (i.e., planning, engaging, executing and evaluating the intervention). Details are provided in Table 2. Some were classic factors that are mentioned in several publications such as the importance of evidence strength and quality, the importance of tailored interventions, or funding. However, one factor could not be included in the CFIR framework because it could not fit in the categories designed by the framework. One publication mentioned that the intervention might have reached a threshold for intervention effectiveness. Indeed it was argued that the reduction of AMU, limitation of AMR prevalence or any other factors that could enhance the fight against AMR might be limited by the currently feasible action in the system.

## Discussion

This study identified characteristics of *E. coli* AMR interventions that affect their implementation in social-ecological AMR systems. We used the AMR-Intervene framework to describe the interventions in their diversity regarding the social-ecological factors of complex systems, as well as reporting quality in scientific publications. The study of 42 unique *E. coli* AMR interventions highlighted a lack of diversity of interventions and their action profile, a lack of reporting regarding implementation strategy, and a need for new, adaptive, flexible and complex interventions to tackle AMR.

### Lack of diversity of interventions

The interventions coded in the database had limited diversity, mainly in their social system and governance. Table 1 highlights the homogeneity within the description of the intervention such as spatial homogeneity, small diversity of actors, target groups and settings, and similar funding sources. All interventions also targeted the individuals (human or animals) and, except for environmental studies, did not target the cell, gene, or ecosystem level. Also, the sector targeted by the intervention was primarily either human or animal health, and One Health interventions remain rare or not reported. Goals and challenges of collective actions are similar and remain in the same categories; typically, one goal and challenge aiming at reducing the state of AMR through surveillance. The homogeneity of the results of the current research underlines the need for more innovation in terms of designing interventions. In the area of governmental action to reduce AMU, Rogers Van Katwyk et al*.* identified 17 types of interventions [25].

Since diversity of action is a key driver of resilience in social-ecological system, this might be an obstacle to building societal resilience to AMR. Moreover, the use of system thinking has highlighted the need for new forms of co-evolutionary governance to tackle AMR [26, 27]. Numerous studies acknowledge the social-ecological context of AMR, its diversity, its co-dependence and co-evolution with our societies, practices, policies, and technologies [11, 19, 28]. In the same way that we increasingly rely on a specific AM to treat an infectious disease, we are using the same type of interventions over and over to respond to the growing AMR crisis. Diversification in actions towards the fight against AMR has been identified as one of the priorities for successful actions [11, 28]. Thus, the lack of diversity is also proof of a lack of understanding and/or acknowledgment of the complexity of the system, which in turn can decrease the power of actions and interventions [18, 29]. More geographical diversity is also desired, as most of the interventions identified were implemented in high-income countries, while AMR interventions in low- and middle- income countries have been rapidly increasing over the last decade [30].

### Black box of intervention implementation

Based on the 52 publications reviewed, it seems that factors related to the implementation, sustainability and success of an intervention are part of a black box [31], i.e., these factors are poorly or not reported in scientific publications. The aim of publishing AMR interventions is obviously to report on what was done, how it was done, and how well it worked. Maximizing the usefulness of past intervention experience requires greater attention to implementation, including the description of the intervention, its implementation strategy, and the sharing of experience with other people involved in implementation [11]. A recent publication stressed the need to include implementation skills in AM stewardship programmes [32]. There is also a need to understand implementation barriers found in different contexts including in low- and middle-income countries [33].

In this narrative review, assessment of the interventions was mainly focused on quantitative indicators, which rationalize and simplify the description process. While this facilitates comparison of intervention results, it obscures the drivers of successes. Furthermore, many different indicators of success or failure were identified in this review. Units and definitions for indicators are not harmonized between evaluations. And still, the main indicators are quantitative, and authors did not explore qualitative methods to assess the interventions. Qualitative assessment of interventions would allow publication of unusual results or feedback about the intervention and things that may not typically be measured in quantitative assessments of interventions (e.g., social capital, acceptability of the intervention) but may be important to intervention success [29]. It could also help assessors identify unexpected consequences of the intervention. We recognize that authors might not yet have the capacity to report the implementation and assessment of interventions for various reasons (e.g., time constraints, publication restrictions, reluctance to use non validated tools), but believe that the involvement of interdisciplinary teams in the design, implementation, evaluation and reporting of interventions can fill this gap.

In most publications we identified a lack of contextualization, as the intervention was not implemented and reported in a global system perspective and was usually seen as an individual action. All readers would benefit from knowing the context, the means, and other details of the intervention [34–38]. Many studies developed frameworks for reporting and/or assessing interventions [39–44], but few included parameters linked to the social-ecological context of interventions, specifically in an AMR system. The scientific literature is in need of a new definition and understanding of AMR interventions, including a global contextualization of it as defined in our study. A new format of publication for health interventions that requires a systematic description followed by criteria to ensure a minimum set of details would be an asset for the scientific community and decision makers [17, 45].

### Limitations—methodology

This study characterized 42 *E. coli* AMR interventions using the AMR-Intervene framework. Several limitations could have influenced the study results. First, coding bias has been largely prevented, but some variables of the framework still rely on subjective assessment by the coder. Therefore, we implemented a versioning system of AMR-Intervene that can be updated depending on research findings and needs of users. Second, the identification of interventions can also be affected by selection bias and affect our capacity to identify them via the online review process. Some interventions might not have clearly mentioned *E. coli* as a targeted pathogen. For example, interventions targeting many pathogens including *E. coli* among others, interventions based on clinical syndromes (e.g., urinary tract infections, frequently caused by *E. coli*), as well as all interventions targeting ESBL (extended spectrum beta-lactamase) resistance. Finally, our study does not avoid the classical publication bias such as the year of publication, the publication of successful interventions only, publication habits and the dominance of high-income countries in the scientific literature. Details of unsuccessful interventions or even failure factors or difficulties that the implementers faced are rarely reported. Thus, this study should be complemented by a parallel survey to directly reach implementers and better capture success and failure factors, non-published or ongoing interventions that are not covered by this study.

Despite these limitations, AMR-Intervene is the first framework built to characterize AMR interventions from a social-ecological perspective. While the framework helps make sense of the variety of interventions targeting AMR among a diversity of contexts worldwide, the present study suggests that the literature focuses on certain types of interventions. Ultimately, we hope that the application of the framework will help to improve how interventions are identified, conceptualized, described, reported, and assessed.

### Towards a better understanding and implementation of AMR interventions

One publication mentioned that activities to fight AMR might have diminishing returns and the reduction of AMU could not be further improved by current means (i.e., AMR interventions as imagined nowadays) and we might need a shift to other more ambitious interventions and/or improve intervention implementation [9].

Improving implementation of interventions is needed to increase our capacity to tackle the global issue of AMR. Indeed, there is a need for interventions that are better adapted to their social-ecological context, interventions that are diverse and flexible, with various angles of attack, at all levels of the social and bio-ecological system. However, if interventions are not detailed and reported in a more systematic manner, we might not have sufficient understanding of the current situation of AMR interventions. Many publications mentioned a lack of data quality and quantity, which was identified as a failure factor of the intervention. A planned and considered data management process would be an asset to any interventions, e.g., include data managers early in the process, identify with stakeholders and partners an appropriate way to collect, transcribe and share the data and information between them.

A successful intervention should also be able to evolve by integrating feedback and adapting to change. Kruk et al. [46] mentioned that interventions should be integrative, adaptive, self-regulating, diverse, and aware. This would lead to a new generation of complex interventions that would improve how we tackle AMR [11]. Diversity in actions and actors may be relevant at the intervention-level, and also at the country or sector-level (e.g., studying interventions from the same sector). One Health interventions and inter and transdisciplinary approaches might help increase the quality of the intervention by addressing the many interlinked aspects between intervention and context. This could result, for example, in organising stakeholder’ meetings to share intervention progress and gain support from the community or government or having better communication about the intervention by fostering participation of the targeted population in the implementation of the intervention. On the other hand, diversity should not compromise the use and application of interventions that are effective and work in different contexts.

Future interventions are also in need of qualitative data about implementation, such as feedback about the experience, success and failure factors, and contextual factors that can impact the effectiveness or continuity of interventions. In this study we gathered a certain number of different success and failure factors, described in the publications. All factors were identified in previous literature but no publications reported factors about the characteristics of individuals involved, the political and economic climate, or the process of executing the intervention among other factors classified by Darmschroder et al*.* [24]. Therefore, there is a need to improve how interventions are assessed, possibly mixing methods of evaluation [29]. While contextual factors cannot always be included in the assessment of interventions, mentioning concomitant interventions, even in a different sector, may help to understand the context. A better understanding and overview of the actions being taken to fight against the AMR issue can also be the aim of a resilient governance system, e.g., listing, gathering, and enhancing AMR interventions in a country.

## Conclusions

The study of *E. coli* AMR interventions identified several factors that can improve the success of interventions when it comes to tackling resistance in this ubiquitous colonizer and pathogen. The study highlighted the lack of diversity in the design and implementation of reported interventions. We clearly identified a lack of One Health interventions, a trend toward interventions with similar goals, governance, and components of response to AMR. It also pointed out that interventions are not designed using a complex system approach. To support adaptation and transformation [47], the fight against AMR is in need of more adaptive, and contextually tailored interventions. We also need to improve how interventions are reported, ideally with guidelines that can be shared among stakeholders in a transparent and comprehensive way. This study should be extended to cover a broader choice of interventions for different pathogens in order to develop an even better understanding and picture of success and failure factors in the battle against AMR.

## Supplementary Information

**Additional file 1.** Online search of E. coli AMR interventions included in the current study via a scoping review.

**Additional file 2.** Supplementary results for the description of articles referring to E. coli AMR interventions, coded in AMR-Intervene.

**Additional file 3.** Supplementary results for the description of E. coli AMR interventions.

**Additional file 4.** Adherence to Reporting Guidelines, the checklist.

## Data Availability

The datasets used and analysed during the current study are available from the corresponding author on reasonable request.

## Abbreviations

*E. coli*: *Escherichia coli*
AM: Antimicrobial
AMR: Antimicrobial resistance
AMU: Antimicrobial use
