# Supplementary material for: Characterizing social-ecological context and success factors of antimicrobial resistance interventions across the One Health spectrum: analysis of 42 interventions targeting E. coli
Source: BMC Infect Dis. 2021 Aug 26;21:873. doi: 10.1186/s12879-021-06483-z (PMC8390193; doi:10.1186/s12879-021-06483-z)
Supplement: Supplementary file 1 — Additional file 1. Online search of E. coli AMR interventions included in the current study via a scoping review. [file 12879_2021_6483_MOESM1_ESM.docx]

Linked to Léger *et al.* **Characterizing social-ecological context and success factors of AMR interventions across the One Health spectrum: Analysis of 42 interventions targeting *E. coli***

# Additional files A: Online search of *E. coli* AMR interventions included in the current study via a scoping review (5 tables)

**Table A1**: Search terms for the literature search of E. coli AMR interventions systematic reviews, validated by the project consortium and used in PubMed database (titles and abstracts only) in June 2018 and 2019

| Category | Keywords, search terms | Year of use |
| --- | --- | --- |
| Population | (“public health" OR "one health" OR "global health" OR “veterinary public health” OR “human health” OR “animal health”) |  |
| Intervention | (Awareness OR training OR education* OR stewardship OR prevention OR intervention OR campaign OR "growth promotion" OR certification OR regulation OR policy OR guideline OR legislation OR surveillance OR benchmarking OR “cross sectional” OR “case control” OR communication OR feedback OR before-after OR “controlled trial” OR “time series studies”) |  |
| Outcome | (antimicrobial* OR antibiotic* OR antiparasitic) AND (resistan* OR use OR usage OR prescrib* OR compliance OR “unexpected consequences” OR “unintended consequences”) |  |
| Publication type of interest | (Review OR “systematic review” OR meta-analysis OR “scoping review” OR Cochrane) | June 2018 |
| Pathogen | *E. coli* OR Escherichia OR Enterobacteriacae | June 2019 |
| Year of publication | 2018 OR 2019 | June 2019 |

**Figure A2:** Simplified flow diagram of systematic reviews to follow the evolution of the number of documents included in the study according to the different steps of review and the source of documentation

|  | 2018 – search of reviews in PubMed | | Other sources | 2019 – search of recent *E. coli* AMR interventions in PubMed |
| --- | --- | --- | --- | --- |
| Total results screened from PubMed | 6898 documents with 38 reviews of AMR interventions identified | |  | 1389 documents |
| Total documents  after *title review* | 391 documents of AMR interventions | 615 documents from reviews of AMR interventions |  | 606 documents |
| Total documents  after *abstract review* | 878 documents | | 45 documents | 97 documents |
| Final number of documents relating an AMR intervention  after *article review* | 594 documents | | | 69 documents |
|  | 663 documents in total | | | |
| Final number of documents relating an *E. coli* AMR intervention | 52 documents | | | |

**Table A3:** Exclusion and inclusion criteria of interventions identified from the online review and research of references among systematic reviews during title and abstract reviews.

| Level of selection | Inclusion criteria | Exclusion criteria |
| --- | --- | --- |
| Title review (first step) | - Paper recommended by the consortium | - Articles not stating at least one of these terms: antimicrobial, antibiotic, drug, resistance. - Policy comparison papers - Recommendation papers and guidelines |
| Abstract review (second step) | - Paper recommended by the consortium | - Theoretical studies with no empirical data presented. - Articles not written in English or French. - Any of the primary exclusion criteria that were not apparent from reading the titles and abstracts only. |

**Table A4**: Eligibility and exclusion criteria of AMR intervention in the third and final stage of selection based on the full article called article review

| Eligibility criteria |  |
| --- | --- |
| Refer to an intervention | Cf. previous definition of an intervention |
| Refer to an AMR intervention | We specifically target interventions that aim to fight against AMR. It can be an intervention acting on the drivers of AMR, altering the pressure points (e.g., AMU), changing the current state (e.g., prevalence of AMR), and/or influencing the impact of AMR (e.g., morbidity due to AMR). We placed no limits based on the intervention type and intent to gather all types of interventions such as specific policies, collaborations, jurisdiction, awareness campaigns, surveillance systems, guidelines for AMU… |
| Written in English or French |  |
|  | no limits placed based on intervention quality |
|  | no limits placed based on paper type quality, study type or quality |
| Exclusion criteria |  |
| Does not meet all eligibility criteria | Paper should meet all eligibility criteria. |
| Studies that have no relevant data presented or obtainable. | We defined ’relevant data’ as an intervention where at least one of the study’s reported outcomes was directly attributable to the implementation of the intervention. |
| Studies that have no real data but use computed data instead | e.g., model description |
| Selection of *E. coli* AMR interventions | |
| *E. coli* | Action(s) of the intervention target(s) specifically *E. coli* or one outcome measurement of the intervention is using *E. coli*. |

**Table A5:** Main exclusion reasons of articles from the AMR interventions pool for the title, abstract, and article reviews.

| Category | Reasons for exclusion |
| --- | --- |
| *E. coli* papers, i.e., papers with intervention description that targets specifically *E. coli* or Enterobacteriaceae or use *E. coli* as an outcome measurement. | Intervention or not, cf. definition |
| Protocol of an intervention with no results or assessment of the intervention (within the paper or not) |  |
| “Surveillance” papers, discrepancy in the definition of surveillance/monitoring | Prevalence survey papers  Use of surveillance data for “research” purpose |
|  | Included in the database: yearly report of a monitoring program if available over several years (potentially in several papers) |
| Risk factor analysis/studies |  |
| Recommendations papers | e.g., state of the situation since many years followed by recommendations and suggestion of national action plan |
| Descriptive studies of the AMR situation in a specific context (e.g., country) |  |
