# Supplementary material for: Characterizing social-ecological context and success factors of antimicrobial resistance interventions across the One Health spectrum: analysis of 42 interventions targeting E. coli
Source: BMC Infect Dis. 2021 Aug 26;21:873. doi: 10.1186/s12879-021-06483-z (PMC8390193; doi:10.1186/s12879-021-06483-z)
Supplement: Supplementary file 2 — Additional file 2. Supplementary results for the description of articles referring to E. coli AMR interventions, coded in AMR-Intervene. [file 12879_2021_6483_MOESM2_ESM.docx]

Linked to Léger *et al.* **Characterizing social-ecological context and success factors of AMR interventions across the One Health spectrum: Analysis of 42 interventions targeting *E. coli***

# Additional files B: Supplementary results for the description of articles referring to *E. coli* AMR interventions, coded in AMR-Intervene (2 tables, 2 figures)

**Table B1:** Summary of the coded articles about the 42 E.coli AMR interventions (n=52 articles coded)

| Publication reference | Name of the intervention | Country of implementation | Level of implementation | Sector of implementation | Challenge of collective action |
| --- | --- | --- | --- | --- | --- |
| (1) | Monitoring of AMR at QECH | Malawi | Local | Human | Surveillance |
| (2) | TRAP | Netherlands | Local | Human | Prevention, Containment |
| (3) | Treatment guideline in ICU | Germany | Local | Human | Conservation |
| (4) | Bundle of interventions in Dutch hospital | Netherlands | Local | Human | Conservation |
| (5) | ASP in Alabama hospital | United States | Local | Human | Conservation |
| (6) | Guideline for rational use in paediatric unit | China | Local | Human | Conservation |
| (7) | Restriction policy in Greek hospital | Greece | Local | Human | Conservation |
| (8) | Stewardship South Korean hospital | South Korea | Local | Human | Surveillance, Conservation |
| (9) | Gentamycin replacement | France | Local | Human | Conservation |
| (10) | ICT Red cross hospital | Japan | Local | Human | Conservation |
| (11) | HCAAS | Taiwan | Local | Human | Conservation |
| (12) | CASRA | China | National | Human | Surveillance |
| (13) | Strama | Sweden | National | Human | Conservation |
| (14) | NARP | Turkey | National | Human | Conservation |
| (15) | Strama | Sweden | Local, National | Human | Surveillance, Conservation |
| (16) | MYSTIC | Belgium, Croatia, Czech Republic, Finland, Germany, Greece, Poland, Russia, Spain, Sweden, Turkey, United Kingdom | Regional | Human | Surveillance |
| (17) | MYSTIC | All countries, Argentina, Australia, Belgium, Brazil, Bulgaria, Canada, Colombia, Croatia, Czech Republic, Finland, Germany, Greece, Hong Kong, Israel, Malta, Mexico, Peru, Poland, Russia, South Africa, Spain, Sweden, Switzerland, Turkey, United Kingdom, United States, Venezuela | International | Human | Surveillance |
| (18) | SARI | Germany | National | Human | Surveillance |
| (19) | SARI | Germany | National | Human | Surveillance |
| (20) | SARI | Germany | National | Human | Surveillance |
| (21) | Voluntary ban of cephalosporin in pigs in Denmark | Denmark | National | Animal | Conservation |
| (22) | MYSTIC | All countries, United States | International | Human | Surveillance |
| (23) | Strama | Sweden | Sub-national, National | Human | Surveillance, Conservation, Innovation |
| (24) | SARI | Germany | National | Human | Surveillance |
| (25) | SARI | Germany | National | Human | Surveillance |
| (26) | DANMAP | Denmark | National | Animal, Food, Human | Surveillance, Conservation |
| (27) | Passive surveillance Ontario - from AHL data | Canada | Sub-national | Animal | Surveillance |
| (28) | Surveillance of *E. coli* in China (pigs and poultry) | China | Sub-national | Animal | Surveillance |
| (29) | CEESA's VetPath programme | Belgium, Czech Republic, Denmark, France, Germany, Italy, Netherlands, Spain, United Kingdom | Regional | Animal | Surveillance |
| (30) | NORM-VET | Norway | National | Animal | Surveillance |
| (31) | RESAPATH | France | National | Animal | Surveillance |
| (32) | Belgian vet monitoring of AMC and AMR | Belgium | National | Animal | Surveillance |
| (33) | CANWARD | Canada | Sub-national, National | Human | Surveillance |
| (34) | LabBase2 | United Kingdom | Sub-national, National | Human | Surveillance |
| (35) | "Raised without AB" beef | United States | Local | Animal | Conservation |
| (36) | SARI | Germany | National | Human | Surveillance |
| (37) | CIPARS | Canada | National | Animal | Surveillance |
| (38) | Voluntary ban of ceftiofur in hatcheries in Japan | Japan | National | Animal | Conservation |
| (39) | Ciprofloxacine generics | Denmark | National | Human | Conservation, Access |
| (40) | Comparison of WWTP methods | Poland | Sub-national | Environment | Prevention, Containment |
| (41) | Surveillance of E.coli bloodstream infections | United Kingdom | Sub-national | Human | Surveillance |
| (42) | Sentinel surveillance for *E. coli* | United Kingdom | National | Human | Surveillance |
| (43) | Research post harvest intervention | United States | Local | Food | Prevention |
| (44) | Voluntary ban of ceftiofur in Québec in poultry sector | Canada | Sub-national | Animal | Conservation |
| (45) | UTIs and AMU | Finland | Sub-national | Human | Conservation, Prevention |
| (46) | Ciprofloxacin restriction | Israel | National | Human | Conservation |
| (47) | Slovenia Multifaceted intervention | Slovenia | National | Human | Conservation |
| (48) | ICARE - phase 2 | United States | Sub-national | Human | Surveillance |
| (49) | NARMS | United States | Sub-national, National | Animal, Food, Human | Surveillance |
| (50) | ISIS-AR | Netherlands | National | Human | Surveillance |
| (51) | Netherlands AMU reduction campaign | Netherlands | National | Animal |  |
| (52) | Strama | Sweden | Local, National | Animal, Human | Surveillance, Conservation |

1. Musicha P, Cornick JE, Bar-Zeev N, French N, Masesa C, Denis B, et al. Trends in antimicrobial resistance in bloodstream infection isolates at a large urban hospital in Malawi (1998-2016): a surveillance study. Lancet Infect Dis. 2017 Oct;17(10):1042–52.

2. Leverstein-van Hall M, Fluit A, Blok H, Box A, Peters E, Weersink A, et al. Control of nosocomial multiresistant Enterobacteriaceae using a temporary restrictive antibiotic agent policy. Eur J Clin Microbiol Infect Dis. 2001;20(11):785–91.

3. Meyer E, Lapatschek M, Bechtold A, Schwarzkopf G, Gastmeier P, Schwab F. Impact of restriction of third generation cephalosporins on the burden of third generation cephalosporin resistant K. pneumoniae and E. coli in an ICU. Intensive Care Med. 2009;35(5):862–70.

4. Willemsen I, Cooper B, Van Buitenen C, Winters M, Andriesse G, Kluytmans J. Improving quinolone use in hospitals by using a bundle of interventions in an interrupted time series analysis. Antimicrob Agents Chemother. 2010;54(9):3763–9.

5. Lee RA, Scully MC, Camins BC, Griffin RL, Kunz DF, Moser SA, et al. Improvement of gram-negative susceptibility to fluoroquinolones after implementation of a pre-authorization policy for fluoroquinolone use: A decade-long experience. Infect Control Hosp Epidemiol. 2018 Dec;39(12):1419–24.

6. Ding H, Yang Y, Wei J, Fan S, Yu S, Yao K, et al. Influencing the use of antibiotics in a Chinese pediatric intensive care unit. Pharm world Sci. 2008;30(6):787–93.

7. Petrikkos G, Markogiannakis A, Papapareskevas J, Daikos GL, Stefanakos G, Zissis NP, et al. Differences in the changes in resistance patterns to third-and fourth-generation cephalosporins and piperacillin/tazobactam among Klebsiella pneumoniae and Escherichia coli clinical isolates following a restriction policy in a Greek tertiary care hospital. Int J Antimicrob Agents. 2007;29(1):34–8.

8. Kim N-H, Han W-D, Song K-H, Seo H, Shin M, Kim TS, et al. Successful containment of carbapenem-resistant Enterobacteriaceae by strict contact precautions without active surveillance. Am J Infect Control. 2014;42(12):1270–3.

9. De Champs C, Franchineau P, Gourgand JM, Loriette Y, Gaulme J, Sirot J. Clinical and bacteriological survey after change in aminoglycoside treatment to control an epidemic of Enterobacter cloacae. J Hosp Infect. 1994;28(3):219–29.

10. Ikeda Y, Mamiya T, Nishiyama H, Narusawa S, Koseki T, Mouri A, et al. A permission system for carbapenem use reduced incidence of drug-resistant bacteria and cost of antimicrobials at a general hospital in japan. Nagoya J Med Sci. 2012;74(1–2):93–104.

11. Chan YY, Lin TY, Huang CT, Deng ST, Wu TL, Leu HS, et al. Implementation and outcomes of a hospital-wide computerised antimicrobial stewardship programme in a large medical centre in Taiwan. Int J Antimicrob Agents. 2011;38(6):486–92.

12. Zou YM, Ma Y, Liu JH, Shi J, Fan T, Shan YY, et al. Trends and correlation of antibacterial usage and bacterial resistance: time series analysis for antibacterial stewardship in a Chinese teaching hospital (2009–2013). Eur J Clin Microbiol Infect Dis. 2015;34(4):795–803.

13. Hanberger H, Skoog G, Ternhag A, Giske CG. Antibiotic consumption and antibiotic stewardship in Swedish hospitals. Ups J Med Sci. 2014;119(2):154–61.

14. Altunsoy A, Aypak C, Azap A, Ergönül Ö, Balik I. The impact of a nationwide antibiotic restriction program on antibiotic usage and resistance against nosocomial pathogens in Turkey. Int J Med Sci. 2011;8(4):339–44.

15. Mölstad S, Löfmark S, Carlin K, Erntell M, Aspevall O, Blad L, et al. Lessons learnt during 20 years of the swedish strategic programme against antibiotic resistance [Programme stratégique suédois contre la résistance aux antibiotiques - 20 années d’enseignements]. Bull World Health Organ. 2017;95(11):764–73.

16. Turner PJ. Meropenem activity against European isolates: report on the MYSTIC (Meropenem Yearly Susceptibility Test Information Collection) 2006 results. Diagn Microbiol Infect Dis. 2008;60(2):185–92.

17. Jones RN, Mendes C, Turner PJ, Masterton R. An overview of the Meropenem Yearly Susceptibility Test Information Collection (MYSTIC) Program: 1997-2004. Diagn Microbiol Infect Dis. 2005;53(4):247–56.

18. Meyer E, Schwab F, Gastmeier P, Rueden H, Daschner FD. Surveillance of antimicrobial use and antimicrobial resistance in German intensive care units (SARI): A summary of the data from 2001 through 2004. Infection. 2006;34(6):303–9.

19. Meyer E, Schwab F, Gastmeier P, Rueden H, Daschner FD, Jonas D. Stenotrophomonas maltophilia and antibiotic use in German intensive care units: data from Project SARI (Surveillance of Antimicrobial Use and Antimicrobial Resistance in German Intensive Care Units). J Hosp Infect. 2006;64(3):238–43.

20. Meyer E, Schwab F, Jonas D, Rueden H, Gastmeier P, Daschner FD. Surveillance of antimicrobial use and antimicrobial resistance in intensive care units (SARI): 1. Antimicrobial use in German intensive care units. Intensive Care Med. 2004;30(6):1089–96.

21. AgersoØ Y, Aarestrup FM. Voluntary ban on cephalosporin use in Danish pig production has effectively reduced extended-spectrum cephalosporinase-producing Escherichia coli in slaughter pigs. J Antimicrob Chemother. 2013;68(3):569–72.

22. Rhomberg PR, Deshpande LM, Kirby JT, Jones RN. Activity of meropenem as serine carbapenemases evolve in US Medical Centers: monitoring report from the MYSTIC Program (2006). Diagn Microbiol Infect Dis. 2007;59(4):425–32.

23. Mölstad S, Erntell M, Hanberger H, Melander E, Norman C, Skoog G, et al. Sustained reduction of antibiotic use and low bacterial resistance: 10-year follow-up of the Swedish Strama programme. Lancet Infect Dis. 2008;8(2):125–32.

24. Meyer E, Schwab F, Jonas D, Ruden H, Gastmeier P, Daschner FD. Temporal changes in bacterial resistance in German intensive care units, 2001-2003: Data from the SARI (surveillance of antimicrobial use and antimicrobial resistance in intensive care units) project. J Hosp Infect. 2005;60(4):348–52.

25. Meyer E, Jonas D, Schwab F, Rueden H, Gastmeier P, Daschner FD. Design of a surveillance system of antibiotic use and bacterial resistance in German intensive care units (SARI). Infection. 2003;31(4):208–15.

26. Hammerum AM, Heuer OE, Emborg HD, Bagger-Skjøt L, Jensen VF, Rogues AM, et al. Danish integrated antimicrobial resistance monitoring and research program. Emerg Infect Dis. 2007;13(11):1632–9.

27. Kadykalo S V, Anderson MEC, Alsop JE. Passive surveillance of antimicrobial resistance in Salmonella and Escherichia coli isolates from Ontario livestock, 2007-2015. Can Vet J = La Rev Vet Can. 2018 Jun;59(6):617–22.

28. Zhang P, Shen Z, Zhang C, Song L, Wang B, Shang J, et al. Surveillance of antimicrobial resistance among Escherichia coli from chicken and swine, China, 2008-2015. Vet Microbiol. 2017 May;203:49–55.

29. de Jong A, Garch F El, Simjee S, Moyaert H, Rose M, Youala M, et al. Monitoring of antimicrobial susceptibility of udder pathogens recovered from cases of clinical mastitis in dairy cows across Europe: VetPath results. Vet Microbiol. 2018 Jan;213:73–81.

30. Kaspersen H, Urdahl AM, Simm R, Slettemeas JS, Lagesen K, Norstrom M. Occurrence of quinolone resistant E. coli originating from different animal species in Norway. Vet Microbiol. 2018 Apr;217:25–31.

31. Boireau C, Morignat E, Cazeau G, Jarrige N, Jouy E, Haenni M, et al. Antimicrobial resistance trends in Escherichia coli isolated from diseased food-producing animals in France: A 14-year period time-series study. Zoonoses Public Health. 2018 Feb;65(1):e86–94.

32. Callens B, Cargnel M, Sarrazin S, Dewulf J, Hoet B, Vermeersch K, et al. Associations between a decreased veterinary antimicrobial use and resistance in commensal Escherichia coli from Belgian livestock species (2011-2015). Prev Vet Med. 2018 Sep;157:50–8.

33. Denisuik AJ, Garbutt LA, Golden AR, Adam HJ, Baxter M, Nichol KA, et al. Antimicrobial-resistant pathogens in Canadian ICUs: results of the CANWARD 2007 to 2016 study. J Antimicrob Chemother. 2018 Nov;

34. Johnson AP. Surveillance of antibiotic resistance. Philos Trans R Soc B Biol Sci. 2015;370(1670).

35. Vikram A, Rovira P, Agga GE, Arthur TM, Bosilevac JM, Wheeler TL, et al. Impact of “Raised Without Antibiotics” Beef Cattle Production Practices On Occurrences of Antimicrobial Resistance. Appl Environ Microbiol. 2017 Sep;

36. Remschmidt C, Schneider S, Meyer E, Schroeren-Boersch B, Gastmeier P, Schwab F. Surveillance of Antibiotic Use and Resistance in Intensive Care Units (SARI). Dtsch Arztebl Int. 2017 Dec;114(50):858–65.

37. MacKinnon MC, Pearl DL, Carson CA, Parmley EJ, McEwen SA. Comparison of annual and regional variation in multidrug resistance using various classification metrics for generic Escherichia coli isolated from chicken abattoir surveillance samples in Canada. Prev Vet Med. 2018 Jun;154:9–17.

38. Hiki M, Kawanishi M, Abo H, Kojima A, Koike R, Hamamoto S, et al. Decreased resistance to broad-spectrum cephalosporin in Escherichia coli from healthy broilers at farms in Japan after voluntary withdrawal of ceftiofur. Foodborne Pathog Dis. 2015;12(7):639–43.

39. Jensen US, Muller A, Brandt CT, Frimodt-Møller N, Hammerum AM, Monnet DL, et al. Effect of generics on price and consumption of ciprofloxacin in primary healthcare: the relationship to increasing resistance. J Antimicrob Chemother. 2010;65(6):1286–91.

40. Korzeniewska E, Harnisz M. Relationship between modification of activated sludge wastewater treatment and changes in antibiotic resistance of bacteria. Sci Total Environ. 2018 Oct;639:304–15.

41. Davies J, Johnson AP, Hope R. Identifying hospital-onset Escherichia coli bacteraemia cases from English mandatory surveillance: the case for applying a two-day post-admission rule. J Hosp Infect. 2017 Nov;97(3):207–11.

42. Abernethy J, Guy R, Sheridan EA, Hopkins S, Kiernan M, Wilcox MH, et al. Epidemiology of Escherichia coli bacteraemia in England: results of an enhanced sentinel surveillance programme. J Hosp Infect. 2017 Apr;95(4):365–75.

43. Dharmarha V, Pulido N, Boyer RR, Pruden A, Strawn LK, Ponder MA. Effect of post-harvest interventions on surficial carrot bacterial community dynamics, pathogen survival, and antibiotic resistance. Int J Food Microbiol. 2019 Feb;291:25–34.

44. Dutil L, Irwin R, Finley R, Ng LK, Avery B, Boerlin P, et al. Ceftiofur resistance in Salmonella enterica serovar Heidelberg from chicken meat and humans, Canada. Emerg Infect Dis. 2010;16(1):48–54.

45. Rummukainen ML, Jakobsson A, Matsinen M, Järvenpää S, Nissinen A, Karppi P, et al. Reduction in inappropriate prevention of urinary tract infections in long-term care facilities. Am J Infect Control [Internet]. 2012;40(8):711–4. Available from: http://dx.doi.org/10.1016/j.ajic.2011.09.013

46. Gottesman BS, Carmeli Y, Shitrit P, Chowers M. Impact of quinolone restriction on resistance patterns of Escherichia coli isolated from urine by culture in a community setting. Clin Infect Dis. 2009;49(6):869–75.

47. Fürst J, Čižman M, Mrak J, Kos D, Campbell S, Coenen S, et al. The influence of a sustained multifaceted approach to improve antibiotic prescribing in Slovenia during the past decade: Findings and implications. Expert Rev Anti Infect Ther. 2015;13(2):279–89.

48. Fridkin SK, Steward CD, Edwards JR, Pryor ER, McGowan Jr JE, Archibald LK, et al. Surveillance of antimicrobial use and antimicrobial resistance in United States hospitals: project ICARE phase 2. Clin Infect Dis. 1999;29(2):245–52.

49. Karp BE, Tate H, Plumblee JR, Dessai U, Whichard JM, Thacker EL, et al. National Antimicrobial Resistance Monitoring System: Two Decades of Advancing Public Health Through Integrated Surveillance of Antimicrobial Resistance. Foodborne Pathog Dis. 2017 Oct;14(10):545–57.

50. der Kuil W, Schoffelen AF, de Greeff SC, Thijsen SFT, Alblas HJ, Notermans DW, et al. National laboratory-based surveillance system for antimicrobial resistance: a successful tool to support the control of antimicrobial resistance in the Netherlands. Eurosurveillance [Internet]. 2017;22(46). Available from: https://www.scopus.com/inward/record.uri?eid=2-s2.0-85034982946&doi=10.2807%2F1560-7917.ES.2017.22.46.17-00062&partnerID=40&md5=fa627714da282a53e18616bcd6566f2a

51. Dorado-García A, Mevius DJ, Jacobs JJH, Van Geijlswijk IM, Mouton JW, Wagenaar JA, et al. Quantitative assessment of antimicrobial resistance in livestock during the course of a nationwide antimicrobial use reduction in the Netherlands. J Antimicrob Chemother. 2016;71(12):3607–19.

52. Mölstad S, Cars O, Struwe J. Strama - A Swedish working model for containment of antibiotic resistance. Eurosurveillance. 2008;13(46):1–4.


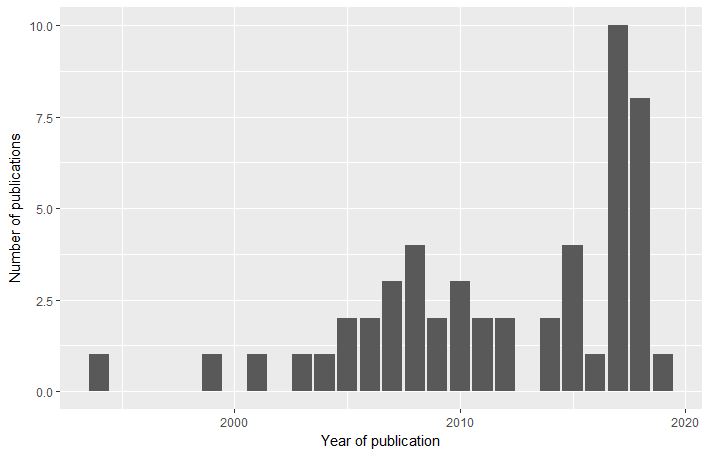


**Figure B2:** Year of publication of the scientific articles describing E. coli AMR interventions coded in AMRIntervene (n=52 articles coded)


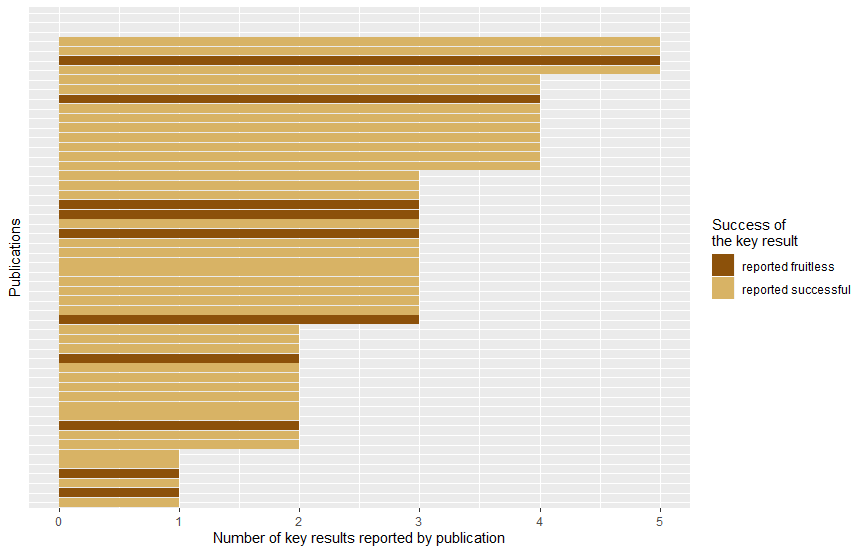


**Figure B3:** Number of key results described in the publications depending on the outcome of the key result of the described E. coli AMR interventions (n=52 articles coded)

**Table B4:** Categorisation of key results identified in the 52 coded E.coli AMR interventions, classified based on the DPSIR framework

| DPSIR category (number of key results identified in the publications) | Description | Sub-category | *Example of key result* |
| --- | --- | --- | --- |
| Driver (3) | Political role of the intervention (policies) |  | *Implementation of new policies for AMR* |
|  | Political role of the intervention (surveillance) |  | *new surveillance system developed* |
| Pressure (37) | Use of AM (27) | Use densities | *Antimicrobial use density of penicillin* |
|  |  | Use/consumption of AM | *Use of AM in intensive care units* |
|  |  | Rate usage and cost/patient day | *rate of AM usage and cost/patient day* |
|  |  | AM treatments | *total number of patients treated with AM* |
|  |  | Prescriptions | *prescription of cephalosporins* |
|  | Sales of AM (1) |  | *AM sales* |
|  | Costs of AM – cost-effectiveness of the intervention (2) |  | *saving in AM sales*  *financial savings in the unit* |
|  | Appropriate use of AM (following of guidelines or not) (4) |  | *appropriateness of AM treatments for community acquired spesis* |
|  | Price of AM (3) |  | *price of ciprofloxacine* |
| Impact (5) | Morbidity (5) | Onset contamination at hospital (nosocomial infections) | *proportion of hospital onset contamination* |
|  |  | Confirmed cases with resistant micro-organism | *recording of new patients infected by GRE* |
| State (59) | Presence of resistance microorganism and identification of AMR genes (28) | Identification of resistant microorganisms (count, incidence) | *Resistance to florfenicol in E. coli streptomycin resistant E. coli* |
|  |  | Identification and/or production of AMR gene | *CA-MRSA genotypes*  *cephalosporins III gen* |
|  | Prevalence of AMR of a specific microorganism (26) | Prevalence | *prevalence E. coli multidrug resistant*  *prevalence of intestinal carriage with GRE* |
|  |  | Rate | *Rate of resistance among selected bacteria* |
|  |  | Proportion between populations | *Proportion of VRE among E. faecium isolate collected* |
|  |  | Densities | *AMR densities for K. pneumoniae and E. coli* |
|  | Incidence of resistant microorganisms (5) |  | *occurrence of QREC in broilers* |
| Directly related to the intervention (32) | Report of results and transparency, publications (8) | Annual report of results (surveillance) | *Annual and three monthly reports of the surveillance results and findings* |
|  |  | Available website | *Website will available results* |
|  |  | Availability of result for actors/stakeholders | *Transparency of results from the first year*  *scientific publication and use of data* |
|  | Delivery of outputs from intervention (e.g., workshop, training, guidelines) (7) |  | *Guidelines and stewardship programs* |
|  | Increase of knowledge and awareness (research results) (6) |  |  |
|  | Cost-effectiveness of the intervention (1) |  |  |
|  | Actors of the intervention and acceptability of the intervention (4) |  | *compliance to the activities from actors* |
|  | Duration of intervention (6) |  | *sustainability of programme more than a decade* |
