# Supplementary material for: Characterizing social-ecological context and success factors of antimicrobial resistance interventions across the One Health spectrum: analysis of 42 interventions targeting E. coli
Source: BMC Infect Dis. 2021 Aug 26;21:873. doi: 10.1186/s12879-021-06483-z (PMC8390193; doi:10.1186/s12879-021-06483-z)
Supplement: Supplementary file 3 — Additional file 3. Supplementary results for the description of E. coli AMR interventions. [file 12879_2021_6483_MOESM3_ESM.docx]

Linked to Léger *et al.* **Characterizing social-ecological context and success factors of AMR interventions across the One Health spectrum: Analysis of 42 interventions targeting *E. coli***

# Additional files C: Supplementary results for the description of *E. coli* AMR interventions (1 table, 5 figures)


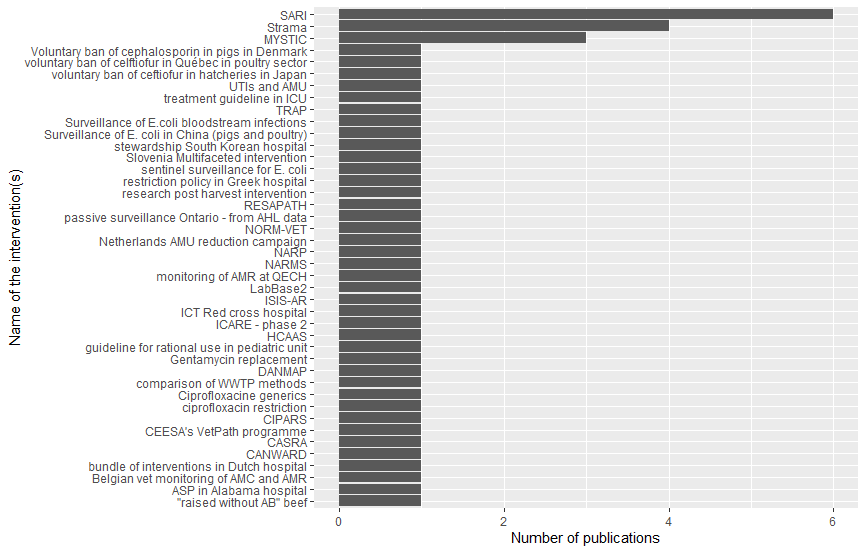


**Figure C1**: Number of interventions coded in AMR-Intervene and the number of articles referring to them (n=52 articles coded)


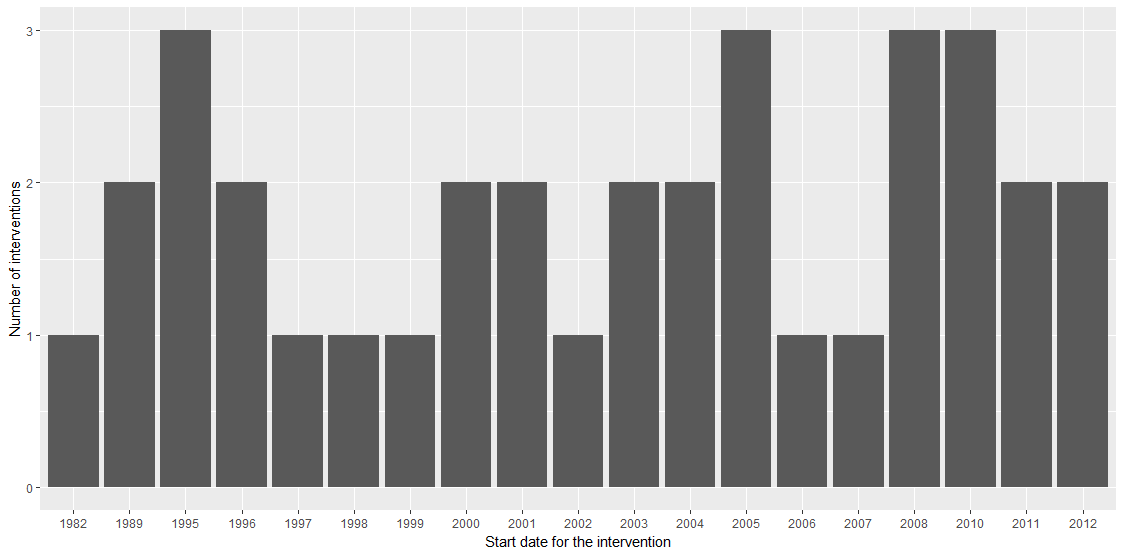


**Figure C2:** Start year of the intervention described in the coded publications (n=42 E. coli AMR interventions from 52 articles coded)


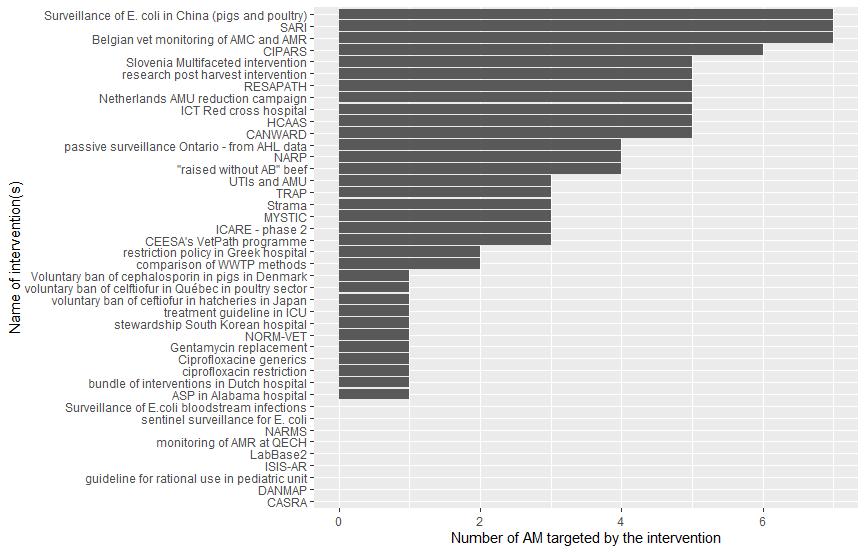


**Figure C3:** Number of AM classes targeted by the E. coli AMR interventions (n=42 E. coli AMR interventions from 52 articles coded)


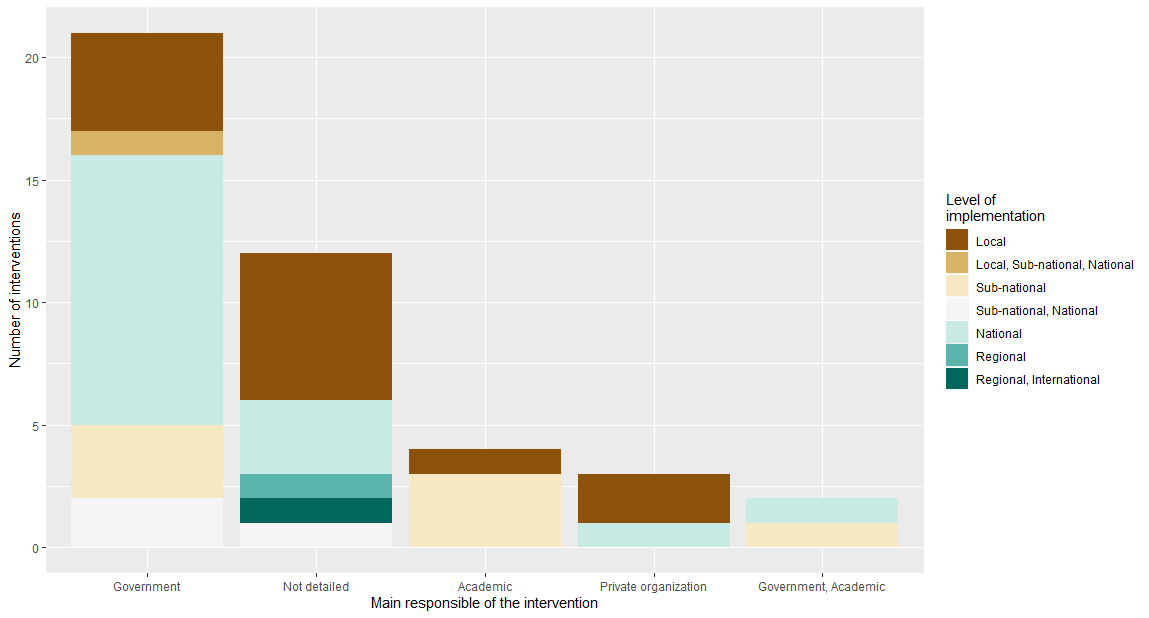


**Figure C4:** Main responsible of the implementation of the E. coli AMR intervention in parallel of the level of implementation of the interventions (n=42 E. coli AMR interventions from 53 articles coded)


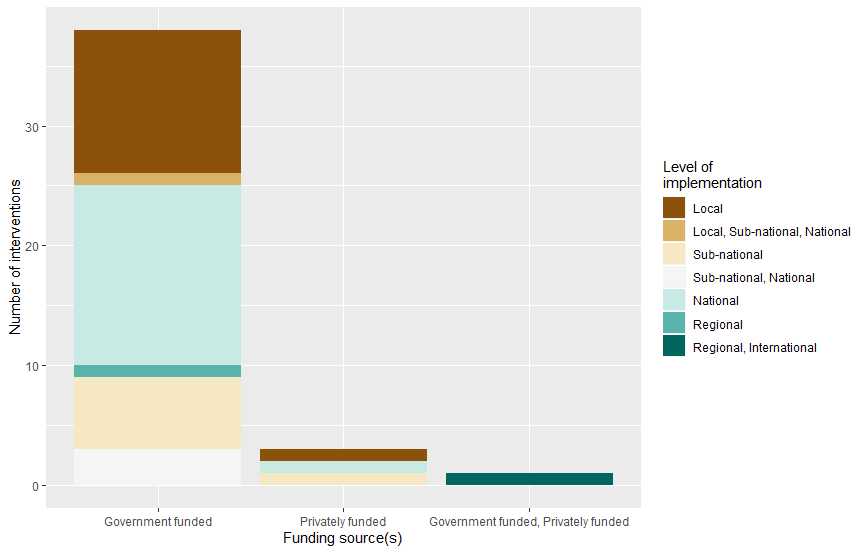


**Figure C5:** Funding body of the intervention depending on the level of implementation of the intervention (n=42 E. coli AMR interventions from 52 articles coded)

**Table C6:** Details of success and failure factors and contextual factors that had an impact on the interventions as described in the 52 studied publications, following the classification of the CFIR, (Consolidated Framework for Implementation Research framework) developped by (1)

| Main domain of CFIR classification | Subdomain of CFIR classification | Description of the factor (success, failure, or contextual factor) | Example in literature (citations) |
| --- | --- | --- | --- |
| Interventions characteristics | Importance of evidence strength and quality. | Data access, management, and analysis (success factor)  *A proper strategy for data access, management, and analysis was identified as a success factor. Data should also be checked for plausibility and completeness. Interventions also mentioned that they do retesting and confirmation of laboratory analyses to increase the data quality.* | *“This integrated program was made possible because access to all relevant data and samples that were already systematically collected from animals, food, and humans has been shared.”* (2) |
|  |  | Number of data (small number of samples)  Quality of the data  Representativeness of the data  Need for long-time data (failure factors)  *Several publications highlighted data limitation as potential failure factors of their intervention, including small number of data/samples, quality of the data (e.g., sampling, recording, entering the data in the database, consistency of data recording, bias in data collection, quality control, semantic homogeneity), low representativeness of the data, and the need for long-time data.* | *“The small number of isolates tested each year may also limit the external validity of the results.* (3)  *Bias should be considered with regard to the following points: ICUs differ according to the number of months they participated. A selection bias is likely because a notable number of resistant strains requested for genotyping was not sent. In addition, misclassification of sensibility testing may bias the resistance data.* (4)  *Representativeness - while this is a substantial number it remains a very small representative samples of the total mastitis pathogen population in the EU and is too small to draw definitive conclusions.* (5)  *The study period after NARP may not be long enough to see the changes in antimicrobial resistance.”* (6) |
|  |  | The lack of adequate literature and knowledge (failure factor)  *Some authors also mentioned the lack of adequate literature and knowledge to help them conducting their research.* | *“At present, there has not been adequate literature to summarize the CASRA, especially using hospital-wide population level data.”* (7) |
|  | Adaptability. | Individually tailored regarding their features and methods. Interventions should also stay flexible and adapt along the intervention. (success factor)  *Interventions were reported more successful if individually tailored regarding their features and methods while remaining flexible to adapt along with the intervention lifecycle. Interventions tailored to their target group (e.g., contact preferences, efficient communication channels) have more chance to be successful. Activities that were adapted or changed as needed during its lifecycle were more likely to be accepted, useful, and successfully achieve intended outcomes. For instance, some interventions changed AMU guidelines, expanded the scope of a surveillance system, and conducted or recommended that interventions be evaluated throughout their lifecycle.* | *“NARMS has adapted to changing needs and threats by expanding the catchment areas for surveillance, examining new isolate sources, adding bacteria under surveillance, adjusting sampling schemes, and modifying antimicrobial agents tested over the years.”* (8) |
|  | Decentralisation of actions and the participation of different actors. | Decentralisation of actions and the participation of different actors. (success factor)  *Decentralising actions to regional and local groups with a diversity and multiplicity of actors was considered to increase the success of the interventions.* | *“More active local interventions appear warranted to facilitate control of this dangerous resistance mechanism.”* (9) |
|  |  | Also, embedding the intervention in pre-existing structures and using existing resources (e.g., sampling strategy already in place) and collaboration with local scientific and political partners may increase the success of the intervention. |  |
|  | Complexity of intervention. | Multimodal interventions. (success factor)  *Multimodal interventions were also reported to increase the success of interventions. For example, one activity such as surveillance of AMU in animal health can be supported by several others like guideline implementation, awareness campaigns, group discussions, media dissemination.* | *“We believe that this new multifaceted approach, which involves local pharmacotherapeutic groups and emphasis on the quality of care, will be effective and will further enhance the quality of care.”* (10) |
|  |  | Benefit of interdisciplinary teams and collaboration between disciplines (success factor)  *Interdisciplinary teams and collaboration between disciplines was identified several times as a catalyst for success. It has been suggested as a factor of success but also an empowering factor for achieving goals. Early involvement of all actors (e.g., stakeholders, multidisciplinary actors) was another supportive action for the intervention.* | *“It was crucial to include a geriatrician in the team. Included expects in infectious diseases and intensive care was really useful, so a wide range of IC issues could be discussed.”* (11) |
|  | Costs. | A sustainable funding was mentioned several times as a fundamental point for success. |  |
| Outer and inner setting | Networks and communication. | Communication with the general public (success factor)  *The communication with the general public, sometimes with the collaboration with media, was a real asset for intervention to increase acceptability but also visibility, transparency, and support. The publications described the use of tools such as a public website, collaboration with national and regional media, available results for specialists and non-specialists, communication of negative effects as well of the intervention.* | *“The MYSTIC Program database, easily accessed via the following website, is regularly updated and available to a wide audience.”* (12) |
|  |  | Communication within the intervention team and actors (success factor)  *Communication within the intervention team and actors was important to encourage the implication and compliance to the intervention. Feedback from actors was also necessary for adapting the intervention if its design did not fit its purpose.* | *“In order to encourage appropriate antimicrobial use, we launched a campaign. At our department, pharmacists started providing important information in the form of drug information.”* (13) |
|  | External policies and incentives. | National level intervention implemented at the same time (contextual factor)  *Interventions at the national level that were implemented concurrently with the studied intervention made an independent assessment of the intervention’s impact difficult, and it might have increased its acceptability/compliance within the target population (e.g., surveillance system and vaccination programme). Concomitant action in a different sector might also influence the targeted sector.* | *“Shortly after the voluntary cessation of all use of cephalosporins in pig production, the Danish government issued a ˜yellow card™” to the pig farmers who had the highest consumption per pig produced. This has led to an overall reduction in use for therapy. Thus, it cannot be excluded that the reduced overall use has also been of importance; however, the most likely explanation for the major reduction in the occurrence of ESC-producing E. coli among Danish pigs is the complete cessation of the use of cephalosporins.”* (14) |
|  |  | Access to healthcare may have an influence in the access of AM, their use, and willingness to people to be treated (contextual factor)  *Access to healthcare was found to have an influence in the access of AMs, their use, and willingness of people to be treated (e.g., lack of health insurance policy).* | *“In the circumstances that reimbursements to hospitals are ultimately proportional to the provided clinical services, it lacks incentive for the hospitals to implement a comprehensive antimicrobial stewardship system in general. In Taiwan, the government adopted a mandatory national health insurance policy in 1995, thereby switching the original free-market medico-economic system to a controlled one, in which the Taiwan Bureau of National Health Insurance becomes the only third-party payer*.” (15) |
|  | Culture. | Background of the country in the fight against AMR (contextual factor)  *A long tradition of fighting AMR in the country was found to make an intervention better accepted by the actors and targeted population. In addition, this historical context was also suggested to show a small impact of the intervention as the previous interventions already improved the quality of life and AMR status of the country. Nonetheless, having functioning institutions in a state, which ensures that compliance with interventions, is an essential cornerstone for limiting AMR.* | *“This could be attributed to the long-term systematic work to maintain appropriate antibiotic prescribing, high use of bacteriological cultures and good compliance with basic hygiene and infection control measures.”* (16) |
| Process | Engaging. | Lack of incentives to participate (failure factor)  *The lack of results was also related to the lack of incentives to participate and follow instructions of the intervention.* | *“In the circumstances that reimbursements to hospitals are ultimately proportional to the provided clinical services, it lacks incentive for the hospitals to implement a comprehensive antimicrobial stewardship system in general.”* (15) |
|  |  | Funding of a specific coordinator or assistant within the intervention (success factor)  *The funding of a specific coordinator or assistant within the intervention was also described as important.* | *“On the basis of these assumptions and the anticipated cost savings, the hospital management funded the project by providing financing for a study coordinator (12 h per week) and a pharmacy assistant (18 h per week) during 2006 and 2007.”* (17) |
|  |  | Sustainable funding (success factor)  *Interventions were identified as costly and require a sustainable financial support to access data and maintain activities.* | *“A sustainable funding is required to allocate time for clinical experts to work closely with prescribers, including audit and feedback to achieve increased adherence to guidelines. A mandate and financial support from the government is needed, as illustrated by the first five years in which the programme lacked funding and was threatened with closure.”* (16) |
|  |  | Direct and strong support from the hierarchy. (success factor)  *Direct and strong support from the hierarchy was mentioned as a factor to enhance success of the intervention by providing leadership and support. Assigning clear responsibilities for the intervention can also contributed to successful intervention implementation.* | *“Implementation had the support of the heads of both departments.”* (18) |
|  | Threshold for intervention effectiveness. | Reached the limits of effectiveness (failure factor)  *Threshold for intervention effectiveness reached was identified as one publication mentioned that the intervention implemented in the country could have reached the limits of effectiveness. Indeed they think that the reduction of AMU, limitation of AMR prevalence or any other factors that could enhance the fight against AMR might be limited by the currently feasible action in the current system.* | *“We believe that audits and intensification of all previous activities have reached their limits of effectiveness, and new activities are needed to further enhance the appropriate use of antibiotics.”* (10) |

1. Damschroder LJ, Aron DC, Keith RE, Kirsh SR, Alexander JA, Lowery JC. Fostering implementation of health services research findings into practice: a consolidated framework for advancing implementation science. Implement Sci. 2009;4(1):1–15.

2. Hammerum AM, Heuer OE, Emborg HD, Bagger-Skjøt L, Jensen VF, Rogues AM, et al. Danish integrated antimicrobial resistance monitoring and research program. Emerg Infect Dis. 2007;13(11):1632–9.

3. Kadykalo S V, Anderson MEC, Alsop JE. Passive surveillance of antimicrobial resistance in Salmonella and Escherichia coli isolates from Ontario livestock, 2007-2015. Can Vet J = La Rev Vet Can. 2018 Jun;59(6):617–22.

4. Meyer E, Jonas D, Schwab F, Rueden H, Gastmeier P, Daschner FD. Design of a surveillance system of antibiotic use and bacterial resistance in German intensive care units (SARI). Infection. 2003;31(4):208–15.

5. de Jong A, Garch F El, Simjee S, Moyaert H, Rose M, Youala M, et al. Monitoring of antimicrobial susceptibility of udder pathogens recovered from cases of clinical mastitis in dairy cows across Europe: VetPath results. Vet Microbiol. 2018 Jan;213:73–81.

6. Altunsoy A, Aypak C, Azap A, Ergönül Ö, Balik I. The impact of a nationwide antibiotic restriction program on antibiotic usage and resistance against nosocomial pathogens in Turkey. Int J Med Sci. 2011;8(4):339–44.

7. Zou YM, Ma Y, Liu JH, Shi J, Fan T, Shan YY, et al. Trends and correlation of antibacterial usage and bacterial resistance: time series analysis for antibacterial stewardship in a Chinese teaching hospital (2009–2013). Eur J Clin Microbiol Infect Dis. 2015;34(4):795–803.

8. Karp BE, Tate H, Plumblee JR, Dessai U, Whichard JM, Thacker EL, et al. National Antimicrobial Resistance Monitoring System: Two Decades of Advancing Public Health Through Integrated Surveillance of Antimicrobial Resistance. Foodborne Pathog Dis. 2017 Oct;14(10):545–57.

9. Rhomberg PR, Deshpande LM, Kirby JT, Jones RN. Activity of meropenem as serine carbapenemases evolve in US Medical Centers: monitoring report from the MYSTIC Program (2006). Diagn Microbiol Infect Dis. 2007;59(4):425–32.

10. Fürst J, Čižman M, Mrak J, Kos D, Campbell S, Coenen S, et al. The influence of a sustained multifaceted approach to improve antibiotic prescribing in Slovenia during the past decade: Findings and implications. Expert Rev Anti Infect Ther. 2015;13(2):279–89.

11. Rummukainen ML, Jakobsson A, Matsinen M, Järvenpää S, Nissinen A, Karppi P, et al. Reduction in inappropriate prevention of urinary tract infections in long-term care facilities. Am J Infect Control [Internet]. 2012;40(8):711–4. Available from: http://dx.doi.org/10.1016/j.ajic.2011.09.013

12. Jones RN, Mendes C, Turner PJ, Masterton R. An overview of the Meropenem Yearly Susceptibility Test Information Collection (MYSTIC) Program: 1997-2004. Diagn Microbiol Infect Dis. 2005;53(4):247–56.

13. Ikeda Y, Mamiya T, Nishiyama H, Narusawa S, Koseki T, Mouri A, et al. A permission system for carbapenem use reduced incidence of drug-resistant bacteria and cost of antimicrobials at a general hospital in japan. Nagoya J Med Sci. 2012;74(1–2):93–104.

14. AgersoØ Y, Aarestrup FM. Voluntary ban on cephalosporin use in Danish pig production has effectively reduced extended-spectrum cephalosporinase-producing Escherichia coli in slaughter pigs. J Antimicrob Chemother. 2013;68(3):569–72.

15. Chan YY, Lin TY, Huang CT, Deng ST, Wu TL, Leu HS, et al. Implementation and outcomes of a hospital-wide computerised antimicrobial stewardship programme in a large medical centre in Taiwan. Int J Antimicrob Agents. 2011;38(6):486–92.

16. Mölstad S, Löfmark S, Carlin K, Erntell M, Aspevall O, Blad L, et al. Lessons learnt during 20 years of the swedish strategic programme against antibiotic resistance [Programme stratégique suédois contre la résistance aux antibiotiques - 20 années d’enseignements]. Bull World Health Organ. 2017;95(11):764–73.

17. Willemsen I, Cooper B, Van Buitenen C, Winters M, Andriesse G, Kluytmans J. Improving quinolone use in hospitals by using a bundle of interventions in an interrupted time series analysis. Antimicrob Agents Chemother. 2010;54(9):3763–9.

18. Meyer E, Lapatschek M, Bechtold A, Schwarzkopf G, Gastmeier P, Schwab F. Impact of restriction of third generation cephalosporins on the burden of third generation cephalosporin resistant K. pneumoniae and E. coli in an ICU. Intensive Care Med. 2009;35(5):862–70.
